# Heparanase is a novel biomarker for immune infiltration and prognosis in breast cancer

**DOI:** 10.18632/aging.203489

**Published:** 2021-08-30

**Authors:** Wen-Jing Yang, Lin Shi, Xiao-Min Wang, Guo-Wang Yang

**Affiliations:** 1 Department of Oncology, Beijing Hospital of Traditional Chinese Medicine, Capital Medical University, Beijing 100010, China

**Keywords:** heparanase, immune infiltration, prognosis, breast cancer

## Abstract

Heparanase (HPSE), an endoglycosidase that cleaves heparan sulfate, regulates a variety of biological processes that promote tumor progression. In this study, we analyzed the correlation between HPSE expression and prognosis in cancer patients, using multiple databases (Oncomine, TIMER, PrognoScan, GEPIA, Kaplan–Meier plotter, miner v4.1, DAVID). HPSE expression was significantly increased in bladder, breast, lung, and stomach cancer compared to matched normal tissues. The increased HPSE expression correlated with poor prognosis and increased immune infiltration levels of B cells, CD8+ and CD4+ T cells, macrophages, neutrophils and dendritic cells in bladder and breast cancer. In breast cancer, the high HPSE expression was associated with basal-like subtypes, younger age (0-40), advanced Scarff-Bloom-Richardson grade, Nottingham Prognostic Index and p53 mutation status. In addition, using a mouse model of breast cancer, our data showed that HPSE upregulated IL-10 expression and promoted macrophage M2 polarization and T cell exhaustion. Together, our data provide a novel immunological perspective on the mechanisms underlying breast cancer progression, and indicate that HPSE may serve as a biomarker for immune infiltration and prognosis in breast cancer.

## INTRODUCTION

Breast cancer is one of the most common tumors and the leading cause of cancer-related deaths in women within the globe [1]. Although improved the strategies for early diagnosis and treatment, the prognosis is still poor, mainly due to inherent aggressive behavior and lack of recognized treatment targets [2]. Therefore, there is an urgent need to develop more sensitive and specific biomarkers for the prognosis of breast cancer patients. In the last 2 decades, immunotherapy including programmed death-1 (PD-1), cytotoxic T lymphocyte associated antigen 4 (CTLA4), and programmed death ligand-1 (PD-L1) inhibitors, demonstrated major breakthroughs and became the major therapeutic approach in solid tumors, such as non-small-cell lung carcinoma (NSCLC) and malignant melanoma [3, 4]. Breast cancer, harboring lots of activated tumor-infiltrating lymphocytes (TILs), is one of the most promising targets of immunotherapy among solid tumors [5–10]. Unfortunately, only a fraction of breast cancer patients respond well to immunotherapy. Since TILs serve as an independent favorable prognostic factor, and a predictive marker of chemotherapy, neoadjuvant therapy, and immunotherapy responses in breast cancer [11–17], identification of specific TILs-associated biomarkers may contribute to development of specific targeted immunotherapies in breast cancer.

The tumor microenvironment (TME), containing tumor cells and non-tumor cells, such as endothelial cells, immune cells, and fibroblasts [18], makes an important impact in tumor metastasis and progression [19–23]. Heparanase (HPSE) is the only mammalian endoglycosidase which can cleaves heparan sulfate (HS), regulates remodeling of the basement membranes and extracellular matrix, as well as promotes the release of many HS-related molecules including cytokines, growth factors, and enzymes. HPSE is upregulated in many types of human tumors [24–27], and this elevation contributes to tumor angiogenesis, growth, metastasis, chemoresistance, and poor prognosis [28–32]. Inhibitors targeting HPSE, such as PI-88 (muparfostat), SST0001 (roneparstat), PG545 (pixatimod), and M-402 (necuparanib) have entered clinical trials. Although the role of HPSE in tumor cells has been well documented, its interaction with non-tumor cells in the TME has not been sufficiently explored. Recent studies have suggested that HPSE interaction with immune cells which contains T cells, B cells, NK cells, macrophages, neutrophils, and dendritic cells, can have both pro- and anti-tumorigenic roles, depending on the setting [33]. In addition, one research has indicated that by increasing HPSE expression in the ILs, tumors can regulate gene expression of many other tumor and non-tumor cells [34]. Thus, analyzing the interaction between HPSE, breast cancer cells, and TILs might show a novel immunological perspective to understand the mechanisms of tumor progression and further improve the clinical practice in breast cancer therapy.

In this research, we analyzed HPSE expression and the role played in the prognosis of cancer patients. In addition, we investigated HPSE association with tumor-infiltrating immune cells and related immune markers in bladder and breast cancer, and analyzed the HPSE correlation with clinicopathological parameters in breast cancer.

## RESULTS

### HPSE mRNA expression in different kinds of human cancer

**Figure 1:**
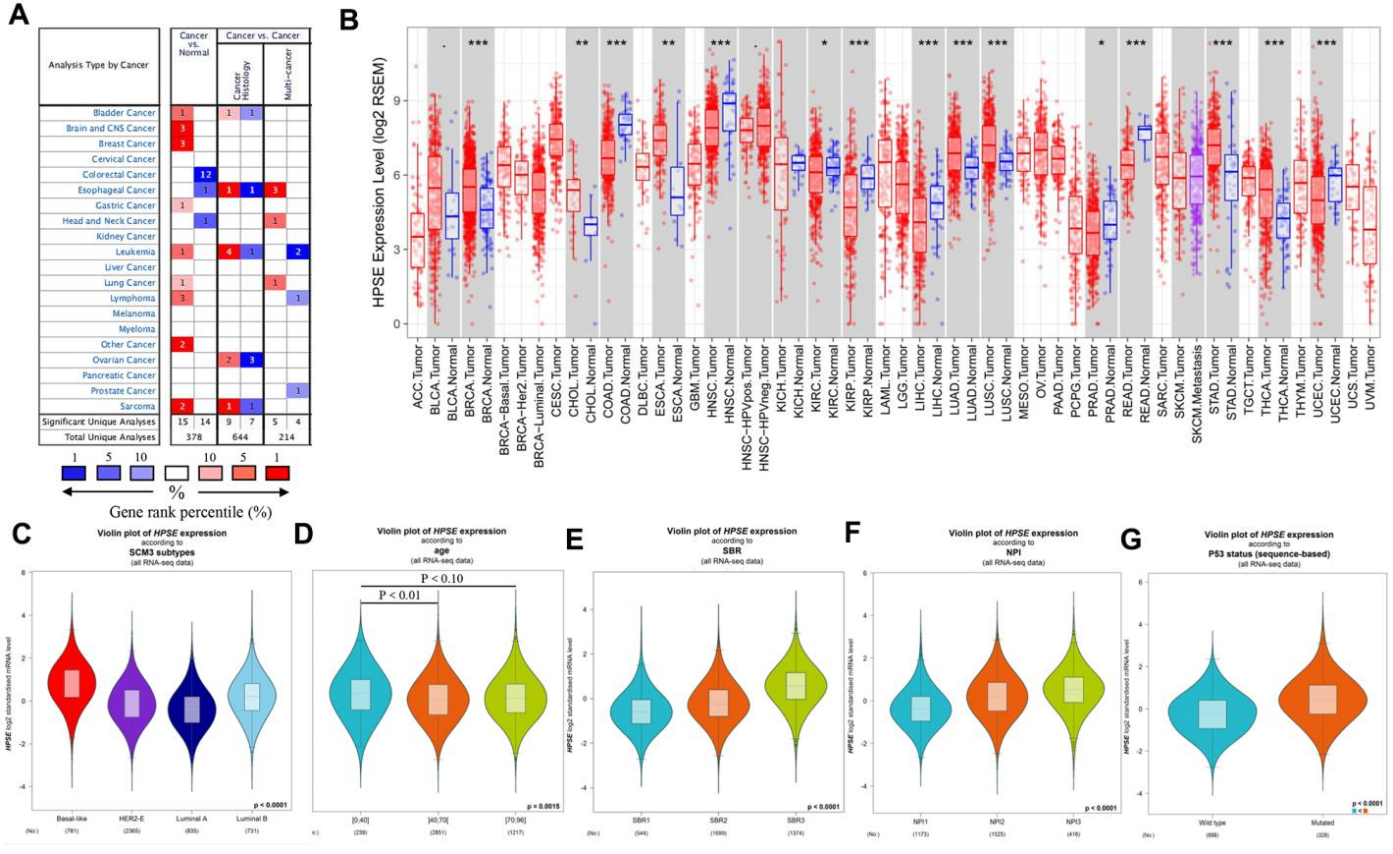
**HPSE expression in different types of human cancers, and relationship between HPSE expression and clinicopathological parameters in breast cancer patients.** (**A**) Increased or decreased HPSE in data sets of different cancers compared with normal tissues in the Oncomine database. The graphic demonstrates the numbers of datasets with statistically upregulated (red) or downregulated (blue) expression of the target mRNA. The grid color is determined by the best gene rank percentile for the analyses within the grid. The Arabic number in each grid represents the number of analyses that met the criteria Gene HPSE. The gene rank was analyzed by the percentile of target genes of HPSE in the top of all genes measured. (**B**) Human HPSE expression in different tumor types in TCGA database determined by TIMER (*P < 0.05, **P < 0.01, ***P < 0.001). The results are shown for the relationship between HPSE expression and SCM3 intrinsic molecular subtype (**C**), age (**D**), SBR (**E**), NPI (**F**), and P53 status (**G**).

To analyze HPSE expression in different kinds of cancer, HPSE mRNA expression in different tumors and matched control tissues were performed using the Oncomine database. The analysis revealed a statistically increased HPSE expression in bladder, brain, CNS, breast, gastric, leukemia, lung, lymphoma, and sarcoma tumors compared to matched normal tissues. However, a decreased HPSE expression was found in colorectal, head and neck, and esophageal cancers (Figure 1A).

To validate the Oncomine results, we analyzed HPSE expression in The Cancer Genome Atlas (TCGA) using the Timer database. As shown in Figure 1B, the HPSE expression was significantly increased in bladder urothelial carcinoma (BLCA), breast invasive carcinoma (BRCA), cholangiocarcinoma (CHOL), esophageal carcinoma (ESCA), lung adenocarcinoma (LUAD), lung squamous carcinoma (LUSC), stomach adenocarcinoma (STAD), and thyroid carcinoma (THCA). In contrast, the HPSE expression was decreased in colon adenocarcinoma (COAD), head and neck cancers (HNSC), kidney renal clear cell carcinoma (KIRC), kidney chromophobe (KICH), liver hepatocellular carcinoma (LIHC), prostate adenocarcinoma (PRAD), rectum adenocarcinoma (READ), and uterine corpus endometrial carcinoma (UCEC).

Comparison of Oncomine and Timer results indicated that the HPSE expression was significantly increased in bladder, breast, lung, and stomach cancer, while it was decreased in colon, head, and neck cancer. Thus, we next analyzed the association of HPSE expression and prognosis in the above cancers.

### High HPSE expression impacts prognosis in bladder and breast cancer

**Figure 2:**
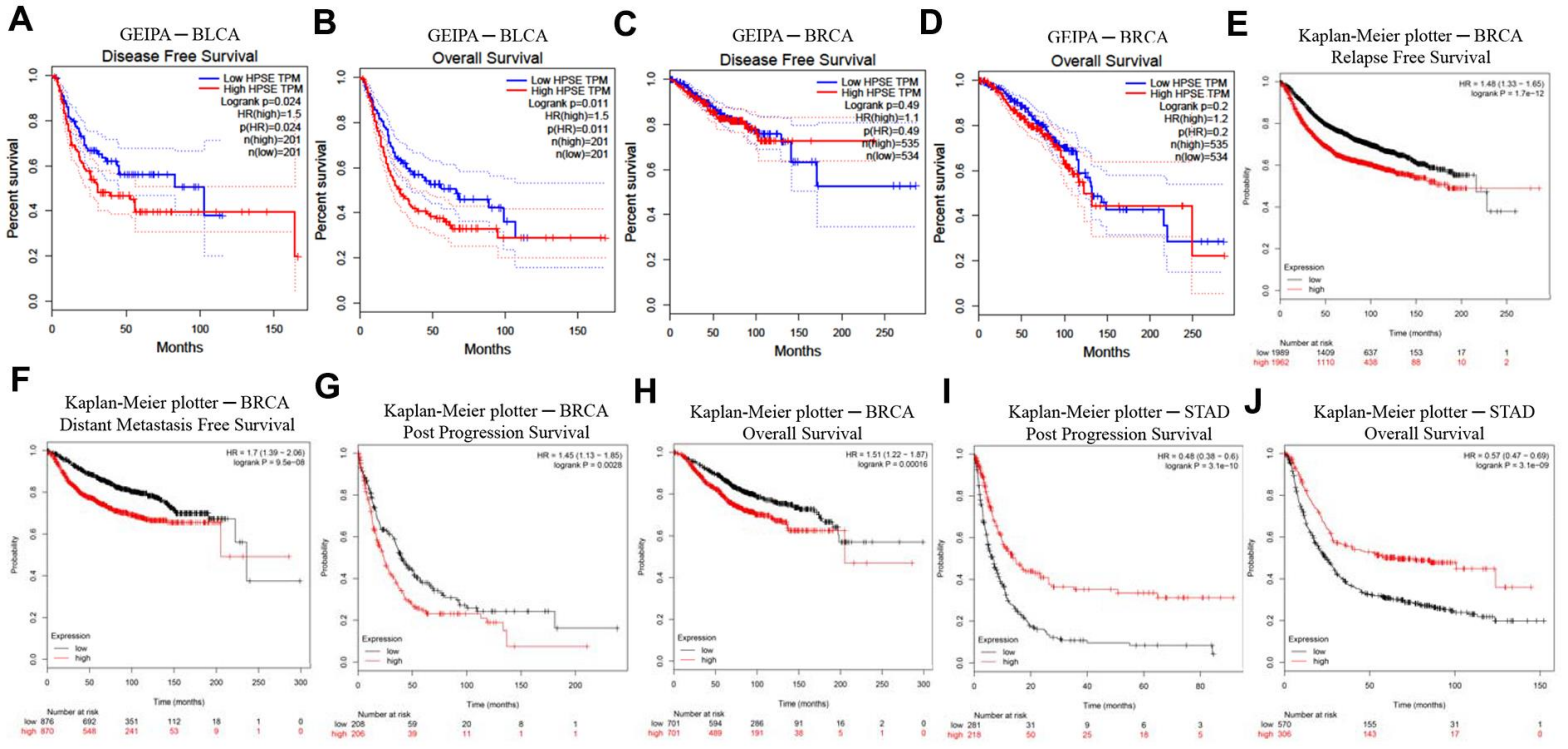
Kaplan–Meier survival curves comparing high and low HPSE expression in different cancer types in GEIPA (**A**–**D**) and Kaplan–Meier plotter (**E**–**J**). (**A**, **B**) Survival curves of DFS and OS in BLCA. (**C**, **D**) Survival curves of DFS and OS in BRCA. (**E**–**H**) Survival curves of RFS, DMFS, PPS and OS in BRCA. (**I**, **J**) Survival curves of PPS and OS in STAD. Bladder urothelial carcinoma (BLCA); breast invasive carcinoma (BRCA); disease-free survival (DFS); distant metastasis-free survival (DMFS); disease-specific survival (DSS); heparanase (HPSE); Gene Expression Profiling Interactive Analysis (GEPIA); overall survival (OS); post progression survival (PPS); relapse-free survival (RFS); stomach adenocarcinoma (STAD).

In order to investigate whether the HPSE expression correlates with prognosis in bladder, breast, gastric, lung, colorectal, head, and neck cancer patients, we used the PrognoScan, GEPIA, and Kaplan–Meier plotter databases to evaluate the impact of HPSE expression on survival. The relationships between HPSE expression and prognosis in different cancers using the PrognoScan database is shown in Table 1. Notably, HPSE expression significantly impacted prognosis in bladder and breast cancers. Analysis of HPSE expression and prognosis in different cancers using the GEIPA database showed that high HPSE expression was related to poor DFS and OS rates in bladder cancer (Figure 2B). Analysis of HPSE expression and cancer prognosis using the Kaplan–Meier plotter database showed that high HPSE expression was related to poor RFS, DMFS, PPS, and OS rates in breast cancer; it was also associated with poor PPS and OS rates in stomach cancer (Figure 2B). Therefore, it is conceivable (confirmed by at least 2 databases) that a high HPSE expression is an independent risk factor, and is associated with poor prognosis in bladder and breast cancer.

**Table 1:** The relationships between HPSE expression and prognosis of different cancers in PrognoScan.

**Dataset**	**Cancer type**	**Endpoint**	**N**	**P-value**	**HR [95% CI]**
GSE13507	Bladder cancer	Disease Specific Survival	165	0.0259141	2.49 [1.12 - 5.56]
GSE1456-GPL96	Breast cancer	Disease Specific Survival	159	0.00755681	1.93 [1.19 - 3.11]
GSE1456-GPL96	Breast cancer	Overall Survival	159	0.0184731	1.60 [1.08 - 2.35]
GSE3494-GPL96	Breast cancer	Disease Specific Survival	236	0.0195956	1.50 [1.07 - 2.11]
GSE4922-GPL96	Breast cancer	Disease Free Survival	249	0.0150854	1.38 [1.06 - 1.79]

### HPSE expression correlates with infiltrating immune cells in bladder and breast cancer

**Figure 3:**
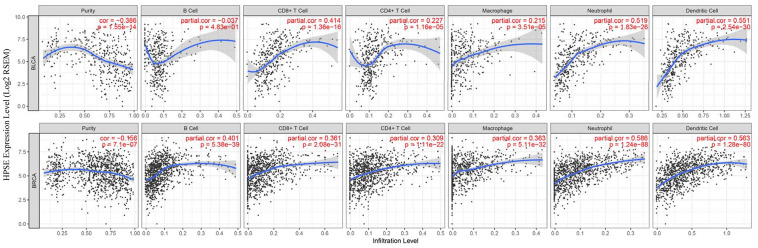
**Correlation of HPSE expression with immune infiltration level in BLCA and BRCA.** In BLCA, HPSE expression has no correlation with infiltrating levels of B cells, medium correlation with infiltrating levels of CD8+ T cells, weak correlation with infiltrating levels of CD4+ T cells and macrophages, and a strong correlation with infiltrating levels of neutrophils and dendritic cells. In BRCA, HPSE expression has medium correlation with infiltrating levels of B cells, CD8+ T cells, CD4+ T cells, and macrophages, and a strong correlation with infiltrating levels of neutrophils and dendritic cells.

TILs are an independent predict factors of survival in cancer. Therefore, we analyzed whether the HPSE expression was associated with immune infiltration levels in bladder and breast cancer. We assessed the correlation of HPSE expression with immune infiltration levels in bladder and breast cancers by the TIMER database. The results showed that in bladder cancer, the HPSE expression is not related to infiltrating levels of B cells, medium correlation with infiltrating levels of CD8+ T cells, weak correlation with infiltrating levels of CD4+ T cells and macrophages, and a strong correlation with infiltrating levels of neutrophils and dendritic cells (DCs). In breast cancer, the HPSE expression showed a medium correlation with infiltrating levels of B cells, CD8+ T cells, CD4+ T cells, and macrophages, and a strong correlation with infiltrating levels of neutrophils and DCs (Figure 3).

To further explore the relationship between HPSE and the infiltrating immune cells, we investigated the association between HPSE and immune cell markers for tumor-associated macrophages (TAM), M1 macrophages, M2 macrophages, monocytes, NK cells, exhausted T cells, Tfh cells, Th1 cells, Th2 cells, Th17 cells, and Treg cells in bladder and breast cancer by the GEPIA database (Table 2). The results showed that in bladder cancer, the HPSE expression had no correlation with Tfh cells, a weak positive correlation with TAM and Treg cells, and a weak negative correlation with M1 macrophages and Th2 cells. The HPSE expression also showed a medium positive correlation with M2 macrophages, monocytes, NK cells, exhausted T cells, and Th1 and Th17 cells in bladder cancer. In breast cancer, the HPSE expression had no correlation with Th2 cells, a weak positive correlation with M1 macrophages, NK cells, and Th17 cells, a medium positive correlation with TAM, exhausted T cells, Tfh cells, Th1 cells, and Treg cells, and a strong positive correlation with M2 macrophages and monocytes.

**Table 2:** Correlation analysis between HPSE and relate genes and markers of immune cells in GEIPA.

**Description**	**Gene makers**	**BLCA**				**BRCA**		
		**R**	**P-value**	**Mean**		**R**	**P-value**	**Mean**
B cell	CD19	-0.012	0.8			0.036	0.23	
	CD79A	-0.0034	0.95			0.055	0.071	
T cell	CD2	0.33	***			0.31	***	
	CD3D	0.22	***			0.18	***	
	CD3E	0.29	***	0.28(***)		0.2	***	0.23(***)
CD8+T	CD8A	0.34	***			0.25	***	
	CD8B	0.042	0.4	0.34(***)		0.25	***	0.25(***)
Dendritic cell	CD1C	-0.012	0.82			0.024	0.43	
	HLA-DPA1	0.31	***			0.39	***	
	HLA-DPB1	0.29	***			0.26	***	
	HLA-DQB1	0.23	***			0.16	***	
	HLA-DRA	0.34	***			0.41	***	
	ITGAX	0.21	***			0.34	***	
	NRP1	0.31	***	0.2817(***)		0.22	***	0.2967(***)
TAM	CCL2	0.21	***			0.28	***	
	CD68	0.46	***			0.52	***	
	IL-10	0.2	***	0.29(***)		0.53	***	0.4433(***)
M1	IRF5	-0.12	0.014			0.34	***	
	NOS2	0.013	0.8			0.082	***	
	PTGS2	0.017	0.73	**-0.12(0.014)**		0.044	0.15	0.211(***)
M2	CD163	0.37	***			0.55	***	
	MS4A4A	0.4	***			0.54	***	
	VSIG4	0.34	***	0.37(***)		0.42	***	0.5033(***)
Monocyte	CD86	0.44	***			0.65	***	
	CSF1R	0.38	***	0.41(***)		0.38	***	0.515(***)
Natural killer cell	KIR2DL1	0.03	0.54			-0.0055	0.86	
	KIR2DL3	0.047	0.34			0.18	***	
	KIR2DL4	0.35	***			0.33	***	
	KIR2DS4	0.03	0.55			0.08	*	
	KIR3DL1	0.071	0.16			0.19	***	
	KIR3DL2	0.088	0.077			0.21	***	
	KIR3DL3	0.035	0.48	0.35(***)		-0.019	0.53	0.198(***)
Neutrophils	CCR7	-0.21	***			0.041	0.180	
	CEACAM8	0.11	0.034			-0.0035	0.91	
	ITGAM	0.2	***	0.0333(***)		0.19	***	0.19(***)
T cell exhaustion	CTLA4	0.35	***			0.38	***	
	GZMB	0.066	0.19			0.35	***	
	HAVCR2	0.47	***			0.6	***	
	LAG3	0.4	***			0.47	***	
	PDCD1	0.27	***	0.3725(***)		0.3	***	0.42(***)
Tfh	BCL6	-0.14	*			0.036	0.240	
	IL21	0.23	***	0.045(***)		0.34	***	0.34(***)
Th1	IFNG	0.32	***			0.38	***	
	STAT1	0.46	***			0.51	***	
	STAT4	0.37	***			0.29	***	
	TBX21	0.28	***			0.31	***	
	TNF	0.26	***	0.338(***)		0.25	***	0.348(***)
Th2	GATA3	-0.33	***			-0.23	***	
	IL13	0.086	0.085			0.092	*	
	STAT5A	0.16	*			0.18	***	
	STAT6	-0.091	0.068	-0.017(***)		-0.0018	*	0.01005(***)
Th17	IL17A	-0.047	0.34			0.079	*	
	STAT3	0.34	***	0.34(***)		0.23	***	0.1545(***)
Treg	CCR8	0.28	***			0.32	***	
	FOXP3	0.36	***			0.33	***	
	STAT5B	0.059	0.23			-0.0053	0.860	
	TGFB1	0.13	0.0084	0.2567(***)		0.061	*	0.237(***)

TAM, Tumor-associated macrophage; Th, T helper cell; Tfh, Follicular helper T cell; Treg, Regulatory T cell; R value of Spearman’s correlation, the absolute value of R, 0-0.09 means no correlation, 0.1-0.3 means weak correlation, 0.3-0.5 means medium correlation, 0.5-1.0 means strong correlation, + means positive correlation, - means negative correlation; MEAN means the average value of relate genes and markers of one specific immune cells with statistical significance. P-value < 0.01 was considered statistical difference, * P-value < 0.01; ** P-value < 0.001; *** P-value < 0.0001.

### HPSE expression correlates with clinicopathological parameters in breast cancer

The correlation between HPSE expression and clinicopathological parameters was explored by the bc-GenExMiner online tool. HPSE expression was compared with different clinicopathological parameters including intrinsic molecular subtype, age, Scarff-Bloom-Richardson (SBR) grade, Nottingham Prognostic Index (NPI), and P53 status. For intrinsic molecular subtypes, the HPSE expression in Basal-like subtype was significantly higher than that in Her2+, Luminal A, and Luminal B subtypes; the HPSE expression in Luminal B subtype was significantly higher than in Her2+ and Luminal A subtypes; and the HPSE expression in Her2+ subtype was significantly higher than in Luminal A subtype (Figure 1C). Regarding age, the HPSE expression in 0-40 years group was significantly higher than in 40-70 and 70-96 years groups; no significant difference was found between 40-70 years group and 70-96 years group (Figure 1D). The SBR histological grade evaluates the degree of duct formation, nucleus pleomorphism, and nuclear division count, while the NPI index stratifies patients into prognostic groups according to lymph node stage, tumor size, and tumor grade. Breast cancer patients with higher SBR grade and NPI tended to express higher levels of HPSE (Figure 1E, 1F). Regarding P53 status, the HPSE expression in mutated group was significantly higher than in wild type group (Figure 1G).

### HPSE expression correlates with M2 macrophage polarization and IL-10 in breast cancer

**Figure 4:**
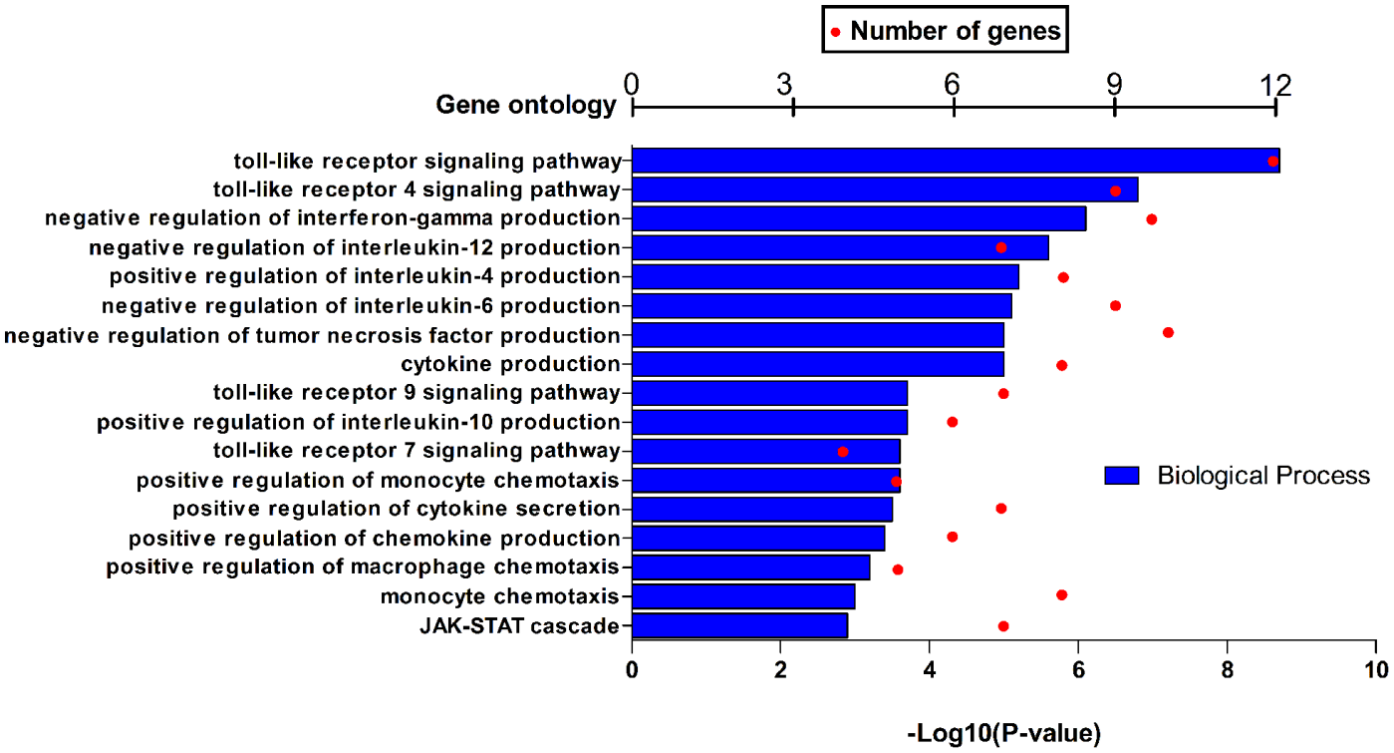
GO function annotations (biological process) for HPSE and target genes in BRCA in DAVID.

HPSE target genes in human breast cancer tissues are listed in Table 3; they are discriminated by R. Using the DAVID software, we found that a total of 17 GO functions (Biological Processes) were enriched (Figure 4); this was supported by analysis of immune markers (Table 4).

**Table 3:** Correlation analysis between HPSE and relate genes and markers of immune cells in breast cancer gene-expression miner v4.1.

**Description**	**Gene makers**	**Basal-Like**			**HER2+**			**Luminal-A**			**Luminal-B**			**TNBC**	
		**R**	**P value**		**R**	**P value**		**R**	**P value**		**R**	**P value**		**R**	**P value**
TAM	CCL2	0.42	***		0.33	*		0.43	***		0.54	***		0.42	***
	CD68	0.48	***		0.4	***		0.38	***		0.39	*		0.51	***
	IL-10	0.52	***		0.44	***		0.61	***		0.54	***		0.51	***
M1	IRF5	0.31	***		0.26	0.0277		0.44	***		0.48	***			***
	NOS2	0.08	0.0553		0.32	*		0.1	0.1526		-0.04	0.7538			*
	PTGS2	-0.1	0.0259		0.04	0.7597		0.26	**		0.26	0.0361			0.2842
M2	CD163	0.61	***		0.52	***		0.64	***		0.65	***		0.67	***
	MS4A4A	0.53	***		0.5	***		0.66	***		0.66	***		0.56	***
	VSIG4	0.4	***		0.4	**		0.56	***		0.56	***		0.46	***
Monocyte	CD86	0.62	***		0.64	***		0.73	***		0.82	***		0.64	***
	CSF1R	0.4	***		0.55	***		0.59	***		0.6	***		0.42	***
T cell exhaustion	CTLA4	0.41	***		0.23	0.0492		0.33	***		0.41	**		0.38	***
	GZMB	0.42	***		0.2	0.0994		0.27	**		0.23	0.0643		0.38	***
	HAVCR2	0.6	***		0.7	***		0.75	***		0.75	***		0.64	***
	LAG3	0.56	***		0.35	0.0024		0.41	***		0.47	***		0.54	***
	PDCD1	0.41	***		0.22	0.0643		0.26	**		0.36	*		0.41	***
Th1	IFNG	0.41	***		0.26	0.0282		0.34	***		0.32	*		0.39	***
	STAT1	0.57	***		0.45	***		0.46	***		0.54	***		0.57	***
	STAT4	0.42	***		0.21	0.0731		0.33	***		0.32	0.0101		0.38	***
	TBX21	0.45	***		0.29	0.0155		0.32	***		0.29	0.0218		0.42	***
	TNF	0.24	***		0.15	0.2001		0.16	0.023		0.35	*		0.34	***

TAM, Tumor-associated macrophage; Th, T helper cell. R value of Spearman’s correlation, the absolute value of R, 0-0.09 means no correlation, 0.1-0.3 means weak correlation, 0.3-0.5 means medium correlation, 0.5-1.0 means strong correlation, + means positive correlation, - means negative correlation. P-value < 0.01 was considered statistical difference, * P-value < 0.01; ** P-value < 0.001; *** P-value < 0.0001.

**Table 4:** HPSE GO function annotations (biological process) for all and intrinsic molecular subtype (RIMSPC) in bc-GenExMiner v4.4.

**Id**	**Description**	**Genes**	**P-value**
**Basal-like**			
GO:0051607	defense response to virus	DTX3L, HERC5, IFIH1, IFIT2, IFIT3, ISG15, OAS1, OAS2, OASL, PLSCR1, RSAD2, RTP4	1.92E-14
GO:0009615	response to virus	CCL8, IFIH1, IFIT2, IFIT3, OAS1, OAS2, OASL, RSAD2	1.34E-10
GO:0060337	type I interferon signaling pathway	IFIT2, IFIT3, ISG15, OAS1, OAS2, OASL, RSAD2	2.27E-10
GO:0045071	negative regulation of viral genome replication	ISG15, OAS1, OASL, PLSCR1, RSAD2	3.82E-08
GO:0060700	regulation of ribonuclease activity	OAS1, OAS2, OASL	5.53E-08
**HER2+**			
GO:0010629	negative regulation of gene expression	CCL3, CCR1, HAVCR2, LGALS9B, LGMN, MSR1	8.44E-06
GO:0030502	negative regulation of bone mineralization	CCL3, CCR1, SRGN	8.71E-06
GO:0042590	antigen processing and presentation of exogenous peptide antigen via MHC class I	FCER1G, IFI30	1.94E-05
GO:0060333	interferon-gamma-mediated signaling pathway	FCGR1B, HLA-DQA2, HLA-H, IFI30	3.46E-05
GO:0006954	inflammatory response	ADORA3, CCL3, CCL8, CCR1, FPR3, HAVCR2, TLR1	3.67E-05
**Luminal A**			
GO:0006955	immune response	C1QC, CCR1, CD86, CTSS, CXCL10, FCGR1B, FCGR2B, FCGR2C, GPR65, HLA-DRA, IGHA1, IGHV3-33, IGHV3-53, IGKV1D-16, IGKV1D-17, IGKV1D-33, IGKV1D-42, IGKV1D-43, IGKV2D-30, IGKV3D-15, IGKV3OR2-268, IGKV5-2, IGKV6D-21, IGSF6, LST1, MARCH1, NCF4, TLR1, TLR4, TLR7, TNFSF13B, TRGC2, TRGV3	6.44E-27
GO:0045087	innate immune response	C1QA, C1QB, C1QC, CD14, CLEC7A, CORO1A, CYBB, FCER1G, HAVCR2, IFI16, IGHA1, IGHV3-33, IGHV3-35, IGHV3-53, IGHV3-74, IGHV3OR15-7, IGHV4OR15-8, LY86, LY96, LYN, MPEG1, NCF1, NCF2, RNASE6, TLR1, TLR4, TLR7, TREM2, TRGV3, TRGV9, TYROBP	3.20E-23
GO:0006911	phagocytosis	AIF1, FCER1G, FCGR2B, IGHA1, IGHV3-33, IGHV3-35, IGHV3-53, IGHV3-74, IGHV3OR15-7, IGHV4OR15-8, ITGB2, MSR1, TREM2	3.49E-14
GO:0006954	inflammatory response	AIF1, C3AR1, CCL8, CCR1, CD14, CLEC7A, CXCL10, CYBB, FCGR2B, FPR3, HAVCR2, HCK, IFI16, ITGB2, LY86, LY96, SIGLEC1, THEMIS2, TLR1, TLR4	1.10E-13
GO:0006958	complement activation	C1QA, C1QB, C1QC, IGHA1, IGHV3-33, IGHV3-35, IGHV3-53, IGHV3-74, IGHV3OR15-7, IGHV4OR15-8, IGKV1D-16, IGKV1D-33, IGKV2D-30, IGKV5-2	2.94E-13
**Luminal B**			
GO:0051607	defense response to virus	CXCL10, GBP1, HERC5, IFI16, IFI44L, IFIH1, IFIT2, IFIT3, MX1, MX2, OAS1, OAS2, OAS3, OASL, PLSCR1, RNASE6, RSAD2, TLR3, TLR7, TRIM22	4.02E-21
GO:0009615	response to virus	CCL8, IFI44, IFIH1, IFIT2, IFIT3, MX1, MX2, OAS1, OAS2, OAS3, OASL, RSAD2, TRIM22	5.31E-15
GO:0006954	inflammatory response	ADORA3, C3AR1, CCL2, CCL8, CCR1, CLEC7A, CXCL10, CXCL11, FPR3, HAVCR2, IFI16, LGALS9, LIPA, NMI, SIGLEC1, THEMIS2, TLR1, TLR4	3.42E-13
GO:0006955	immune response	CCR1, CD80, CD86, CTSC, CTSS, CXCL10, CXCL11, FCGR1B, FCGR2C, GPR65, IFI44, IFI44L, IGLV6-57, MARCH1, TLR1, TLR4, TLR7, TNFSF13B, TRIM22	5.83E-13
GO:0060337	type I interferon signaling pathway	IFIT2, IFIT3, MX1, MX2, OAS1, OAS2, OAS3, OASL, RSAD2	4.89E-11

**Figure 5:**
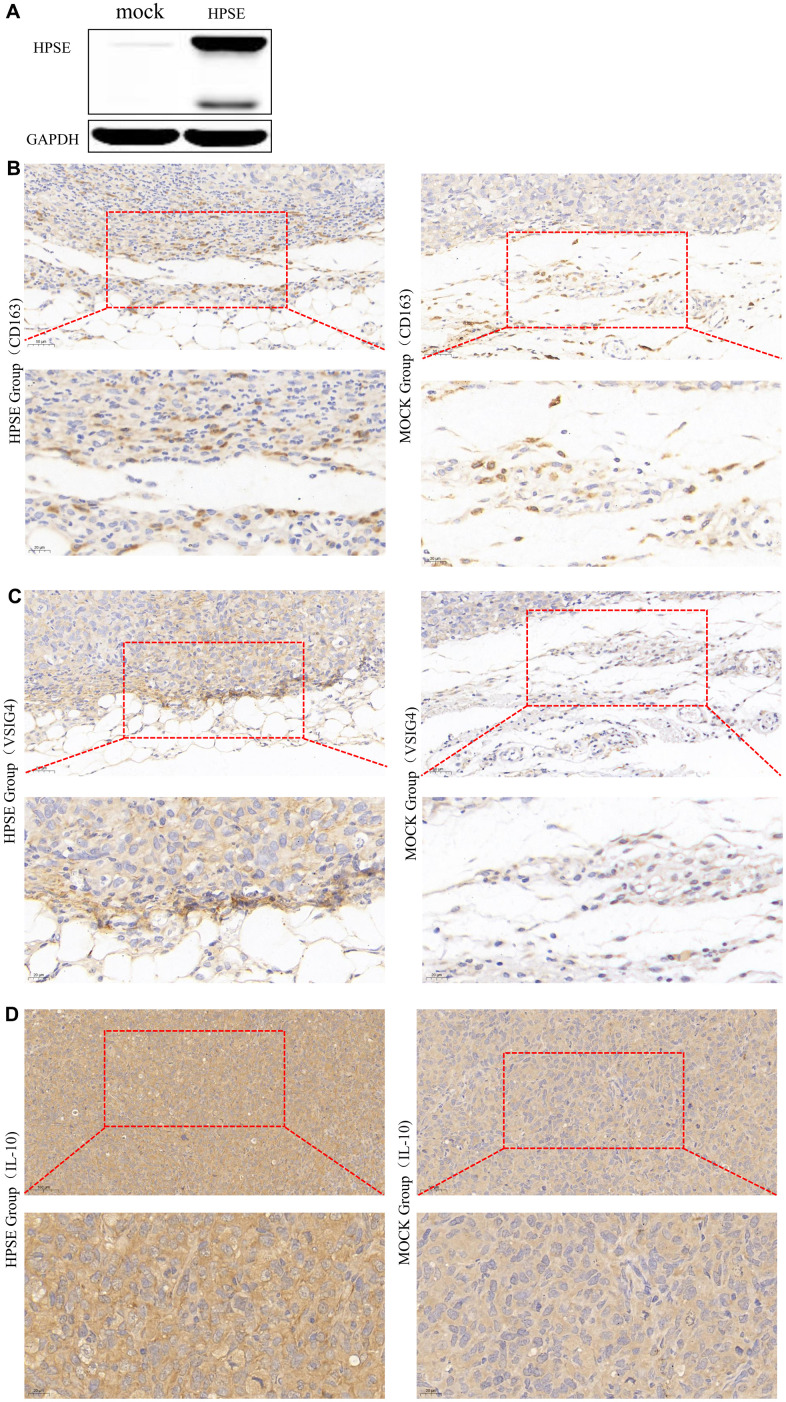
HPSE expression between MDA-MB-231-HPSE and MDA-MB-231-mock cells (**A**). HPSE expression correlates with IL-10 induced M2 macrophage polarization in a mouse breast tumor model: IHC staining of (**B**) CD163, (**C**) VSIG4, and (**D**) IL-10.

Next, using Human MDA-MB-231-HPSE and MDA-MB-231-mock cells which with high and low expression of HPSE (Figure 5A), we validated our data in a mouse model of breast cancer, using mice overexpressing HPSE and a control mock group. Because macrophages usually infiltrate at the edges of tumor tissues, we analyzed the infiltration of macrophages by immunohistochemical staining (IHC) at the tumor tissue edges. IHC staining showed that the HPSE expression correlated with an increased expression of CD163 (Figure 5B) and VSIG4 (Figure 5C), which are markers of M2 macrophages. Compared with MOCK group, M2 macrophages in HPSE group tended to infiltrate into tumor tissues. In addition, the HPSE expression related to an increased expression of IL-10 (Figure 5D), which is known to induce macrophage polarization into the M2 phenotype.

## DISCUSSION

HPSE, the only mammalian endoglycosidase that cleaves HS, contributes to tumor angiogenesis, growth, metastasis, chemoresistance, and poor prognosis in multiple tumors [24–32]. In this research, we demonstrate that the HPSE expression is obviously increased in bladder, breast, lung, and stomach cancer, but decreased in colon, head, and neck cancer. In addition, our data show that the high HPSE expression is an independent risk factor in bladder and breast cancer, indicating that HPSE can be used as a prognostic biomarker for bladder and breast cancer. These findings are supported by previous studies demonstrating that high HPSE gene and protein levels are associated with bladder cancer invasion and metastasis [35]. Analysis of HPSE in urine from 282 individuals showed that the urine HPSE levels were elevated during bladder cancer progression [36]. HPSE mRNA levels in bladder cancer tissues related to tumor stage, histological grade, size, number, recurrence and lymph node metastasis [37]. Inhibition of HPSE expression suppressed invasion, migration and adhesion of bladder cancer cells [38]. In addition, HPSE overexpression accelerated the obesity-associated breast cancer progression [39]. However, the HPSE expression in metastatic lesions does not always reflect the expression in primary tumors. In stage I breast cancer patients, a strong HPSE staining was associated with shorter overall survival rates [40]. Tumor growth, vascularization and recurrence were significantly reduced by inhibition the procoagulant activity of HPSE [41]. Furthermore, inhibition of HPSE expression reduced tumor metastasis by reducing extracellular regulated protein kinase (ERK) and phosphorylation of protein kinase B (Akt) [42].

The immune cells in TME play a critical role in tumor progression, and are recognized as an independent predict factor of cancer survival. Moreover, the immune cells- associated HPSE has important pro-tumorigenic and anti-tumorigenic functions [33]. A previous study has indicated that the increased HPSE expression in TILs can regulate gene expression in other tumor and non-tumor cells [34], suggesting that there might be an HPSE-TME crosstalk that can affect occurrence, development, and fate of tumors. Our study proved the correlation between HPSE expression and tumor-infiltrating immune cells, and supported the intimate correlation between HPSE expression and immune cell infiltration in bladder and breast cancer.

TAMs are the most plentiful immune cells within the TME [43, 44]. According to their function, macrophages can be divided into M1 and M2 phenotypes. The M1 phenotype is characterized by pro-inflammatory cytokines (IL-1β, TNFα, INFα, IL-6, IL-12, IL-16) and tumoricidal activity. In contrast, the M2 phenotype is characterized by anti-inflammatory cytokines (IL-4, IL-10, IL-13, TGF-β) that promote angiogenesis, tissue remodeling, and repair [45, 46]. Therefore, macrophages play a dual role in tumor growth: on one hand, initiating immune responses against transformed cells; and on the other hand, promoting tumor angiogenesis and growth [47–50]. Our data showed that HPSE expression had no or weak correlation with the markers of M1 macrophage, such as NOS2, PTGS2, and IRF5, while HPSE expression had medium or strong correlation with M2 macrophage markers, such as CD163, VSIG4, and MS4A4A. In addition, IL10, a crucial anti-inflammatory cytokine that regulates M2 macrophage polarization, had a strong positive correlation with HPSE expression, indicating that HPSE overexpression might regulate the IL10-mediated M2 macrophage polarization. Our results indicated that HPSE might activate Tregs (CCR8, FOXP3, and TGFB1). In addition, HAVCR2 (TIM-3), a crucial gene that regulates T cell exhaustion, positively correlated with HPSE expression, suggesting that HPSE expression might regulate the TIM-3 mediated T cell exhaustion. These results demonstrated the important role of HPSE in regulating recruitment and activity of immune infiltrating cells in breast cancer, and suggested that HPSE might regulate immune escape in the breast cancer microenvironment.

Macrophages are often found both in primary and metastatic tumors, and contribute to tumor progression [51, 52]. In HPSE knockout mice, macrophages decreased the levels of pro-inflammatory cytokines (TNF-α, IL-1β, CXCL2, and IL6), and impaired infiltration into lung tumors, which were smaller than tumors in wild type animals [53]. In a mouse model of pancreatic cancer, HPSE overexpression was associated with increased macrophage expression of M2 cytokines IL-6, IL-10, CCL-2, VEGF, and macrophage scavenger receptor-2 (MSR-2), increased tumor size, and increased levels of tumor-infiltrating macrophages [54]. In addition, increased HPSE expression in epithelium has been associated with inflammation and inflammation-associated tumorigenesis, such as inflammatory bowel disease (IBD) [55], pancreatitis [56], and esophageal carcinoma [57]. HPSE has been also suggested as a key regulator of the tumor microenvironment [53] and tumor progression [58]. These findings show that HPSE, regardless of its cellular source, promotes tumorigenesis.

Inflammation-induced HPSE is involved in coupling inflammation and cancer [53]. Toll-like receptors (TLRs) lie upstream of the signaling cascade that leads to cytokine induction by HPSE [59, 60]. HPSE is required for macrophage activation, crosstalk with the tumor microenvironment, and tumorigenesis; the mechanism involves HPSE-mediated TLR activation at the cell membrane, followed by Erk/p38/JNK activation and AP1-mediated transcription [61]. However, the exact mechanism of how HPSE regulates the macrophage phenotype is not understood. Our data indicate that HPSE participates in TLR signaling and JAK-STAT cascade by promoting cytokine production. Specifically, HPSE seems to promote macrophage polarization towards the M2 phenotype by suppressing interferon-gamma, IL-12, and IL-6 production, and upregulating IL-4 and IL-10 production [62–64]. In addition, our clinicopathological parameter analysis indicated that HPSE overexpression is associated with basal-like subtypes, younger age (0-40), advanced SBR grade, advanced NPI grade, and P53 mutated status.

Our previous bioinformatics and experimental studies demonstrated that HPSE promotes malignant progression, angiogenesis and metastasis in breast cancer by enhancing the crosstalk between tumor cells and platelet [65]. In this study, multi-database integration analysis indicated that high HPSE expression contributes to macrophage M2 polarization and T cell exhaustion, thus promoting tumor growth. These findings were corroborated also by IHC staining of breast tumor tissues; however, since the nude mouse model is not suitable for T cell analysis, we focused on M2 macrophage polarization. The results indicated that the protein levels of IL-10, CD163, and VSIG4 were significantly increased in breast tumor tissues in mice overexpressing HPSE, indicating that HPSE might promote macrophage M2 polarization (CD163, VSIG4) by upregulating IL-10. Together, our results show that HPSE may serve as a novel biomarker for immune infiltration and prognosis in breast cancer.

## MATERIALS AND METHODS

### Analysis of the expression of HPSE

### Oncomine database analysis

The Oncomine database (http://www.oncomine.org) [66, 67] collects transcriptomic cancer data for biomedical study. Using the Oncomine database, the HPSE expression was compared between cancer tissues and their matched normal tissues. The threshold was: p-value ≤ 1E-4, fold change ≥ 2, and gene rank ≥ top 10%.

### TIMER database analysis

TIMER is a database incorporating 10009 samples with 23 cancer types based on TCGA (https://cistrome.shinyapps.io/timer/); HPSE expression in various cancers was compared between cancer tissues and their matched normal tissues.

### HPSE and clinical prognosis

### PrognoScan database analysis

The PrognoScan (http://www.abren.net/PrognoScan/) is an online database used to evaluate the biological relationship between gene expression and prognostic contains overall survival (OS), relapse-free survival (RFS), distant metastasis-free survival (DMFS), disease-specific survival (DSS), and disease-free survival (DFS) in various types of cancers [68], and provide corresponding p-value, hazard ratio (HR), and 95% confidence intervals. Therefore, it has been used to analyze the correlation between the expression of HPSE and survival in different cancers with the adjusted cox p-value < 0.05.

### GEPIA database analysis

Gene Expression Profiling Interactive Analysis (GEPIA) (http://gepia.cancer-pku.cn/index.html) is used to perform survival analysis (OS and DFS) depended on RNA sequencing data from TCGA database [69]. And in our study, was used to analyze correlation between HPSE mRNA expression and survival in various types of cancers; HR and log-rank p-values were provided, and the threshold was p-value < 0.05.

### Kaplan–Meier plotter database analysis

Kaplan–Meier plotter database (http://kmplot.com/analysis/) is used for analyzing gene association with OS, RFS, DMFS, and post progression survival (PPS) in breast, ovarian, lung and gastric cancer [70]. And in our study, was used to identify the correlation between HPSE mRNA expression and survival in the above four cancer types. The HR and log-rank p-values were provided, and the threshold was p-value < 0.05.

### HPSE and infiltrating immune cells and markers

### TIMER database analysis

TIMER (https://cistrome.shinyapps.io/timer/) using deconvolution statistical method to analyze the infiltration levels of immune cells including B cells, CD4+ T cells, CD8+ T cells, neutrophils, macrophages, and dendritic cells (DCs) based on gene expression profiles [71]. And in our study, was used to analyze the correlation between HPSE expression and the above immune infiltrating cells in bladder and breast cancers, and provided partial correlation coefficients; the threshold was p-value < 0.01. The absolute value of R, 0-0.09 meant no correlation, 0.1-0.3 meant weak correlation, 0.3-0.5 meant medium correlation, and 0.5-1.0 meant strong correlation.

### GEPIA database analysis

We used GEPIA database to identify the correlation between HPSE and related genes and markers in immune cells. the threshold was p-value < 0.01; R, 0-0.09 meant no correlation, 0.1-0.3 meant weak correlation, 0.3-0.5 meant medium correlation, and 0.5-1.0 meant strong correlation.

### HPSE in breast cancer

### Breast cancer gene-expression miner v4.1 database analysis

Breast Cancer Gene-Expression Miner v4.1 (bcGenExMiner v4.1) [72, 73], was utilized to evaluate the correlation between HPSE expression and clinicopathological parameters in breast cancer.

### GO functional annotation analysis

The database for annotation, visualization, and integrated discovery (DAVID) v.6.8 (https://david.ncifcrf.gov) [74] was used to perform GO [75] functional annotation analyses (positive target genes of HPSE). The background list parameter was human genome, and the threshold was p-value < 0.05.

### *In vivo* studies

Human MDA-MB-231-HPSE and MDA-MB-231-mock cells, which represented breast cancer cell lines with high and low expression of HPSE, were handseled by Dr. Israel Vlodavsky, the GFP inserts were performed in Glyconovo Technologies Co., Ltd, and the HPSE expression have been detected between MDA-MB-231-HPSE and MDA-MB-231-mock cells by Western blot analysis, and the specific operation method were described previously [65].

Cell culture, animal care, and establishment of a nude mouse model were described previously [65].

The breast tissues were fixed by immersing them in 10% neutral buffered formalin at room temperature for 24 h, and the paraffin embedding process was performed. Cut the paraffin block of tumor tissue into 5μm sections for IHC staining: The tissue slides were dewaxed in xylene and rehydrated in a graded alcohol bath. Slides were immersed into EDTA antigen extraction buffer and microwaved, and then treated with 3% hydrogen peroxide in methanol to quench the activity of endogenous peroxidase, and combined with 3% bovine serum white, the proteins are incubated together to block non-specific binding. Mouse anti-CD163 (1:100; 93498S, CST), anti-IL-10 (1:100; ab34843, Abcam), and anti-VSIG4 (1:100; PA5-52018, Thermo) antibodies were incubated overnight at 4° C, and then incubation with horseradish-peroxidase (HRP)-conjugated secondary antibody. Expression of CD163, IL-10, and VSIG4 in tissues was assessed by two pathologists. The staining outcomes were assessed as the intensity on a scale of 0 to 3; 0 (no staining), 1 (weak staining), 2 (moderate staining), and 3 (strong staining). Positive tumor cell percentage was semi-quantitatively assessed on a scale of 0 to 4; 0 (none), 1 (1–25%), 2 (26–50%), 3 (51–75%), and 4 (>75%). Histochemical score (H-score) of staining was calculated by multiplying these two variables.
